# Uniting minds and methods: how interprofessional education advances male infertility research

**DOI:** 10.17179/excli2023-6641

**Published:** 2024-01-12

**Authors:** Pallav Sengupta, Sulagna Dutta

**Affiliations:** 1 Physiology, Department of Biomedical Sciences, College of Medicine, Gulf Medical University, Ajman, UAE; 2 School of Life Sciences, Manipal Academy of Higher Education (MAHE), Dubai, UAE

## ⁯

Male infertility is a prominent global health issue, affecting countless couples striving for conception. Although female infertility often garners more attention, it is important to recognize that male-factor infertility contributes to nearly 50 % of the global infertility cases (Agarwal et al., 2015[1]). This highlights the urgent need to deepen our understanding and devise effective strategies to address male reproductive challenges. A considerable number of these cases are labeled as '*idiopathic*' or '*unexplained*' (Sengupta et al., 2022[7]). This ambiguity impedes the development of targeted therapies, often leading to broad-spectrum treatments that may not cater to the unique needs of each affected individual. This scenario suggests that the care for male infertility may not always receive the focused consideration required. Conventional clinical approaches may fall short in grappling with the intricate mechanisms of male reproductive disorders. Here, the role of Interprofessional Education (IPE) becomes paramount. IPE, championing a collaborative learning approach among varied medical disciplines (Thistlethwaite, 2012[9]), offers a comprehensive lens to view male infertility. Collaboration among urologists, endocrinologists, geneticists, and reproductive biologists can significantly enhance the detection of hidden causative mechanisms. 

Male infertility can stem from a variety of sources, including hormonal disparities, anatomical obstructions, genetic determinants, and lifestyle choices (Poongothai et al., 2009[5]; Sengupta and Banerjee, 2014[6]; Sengupta et al., 2022[7]). Considering this wide range, adopting a multidisciplinary methodology is essential. Professionals such as urologists, endocrinologists, geneticists, and mental health specialists each offer a unique perspective on the issue. For example, while a urologist might identify anatomical issues, a geneticist could highlight hereditary conditions that result in infertility. Moreover, lifestyle plays a critical role, with elements like stress, tobacco use, and obesity being linked to diminished sperm quality. This collective knowledge facilitates the formulation of advanced management plans, ensuring men facing infertility get the best possible care. In the global quest to tackle infertility challenges more adeptly, interprofessional collaborations are bound to be instrumental in enriching our understanding and interventions for male infertility. 

The World Health Organization (WHO) defines IPE as an approach where individuals from at least two professions acquire knowledge about, from, and alongside each other, enhancing collaborative efforts and improving healthcare results (WHO, 2013[10]). IPE encourages collaborative learning among multiple professions, emphasizing mutual understanding and shared knowledge (Thistlethwaite, 2012[9]). This culture of interprofessional respect eradicates professional barriers and prioritizes comprehensive patient care. Implementing IPE in male infertility research provides a stage for specialists from varied backgrounds to collaboratively tackle the challenge, leveraging their distinct proficiencies (Buring et al., 2009[2]). Such an approach has multiple benefits like enhanced clinical understanding, holistic patient care and accelerated research.

IPE offers significant potential, yet its implementation faces certain obstacles. Various professions come with their distinct terminologies, methodologies, and protocols (Olenick et al., 2010[4]). To achieve fluid communication and collaboration, dedicated endeavors are essential. To promote interprofessional synergy, institutions can adopt developing Shared Curriculums, promoting collaborative research and organizing interdisciplinary conferences (Thannhauser et al., 2010[8]). To clarify the significance of this pedagogical approach, a valid example of IPE application can be considered. For example, applying IPE to the field of viral-infection-mediated male infertility may present a potent strategy for uncovering the underlying causative mechanisms and designing specific treatments. This approach is rooted in a multidisciplinary method, where male infertility caused by viral infections, being complex and multi-factorial, can be thoroughly analyzed through the integration of expertise from virologists, andrologists, reproductive biologists, urologists, geneticists, and other professionals. It hinges on precision in early diagnosis by identifying specific viral attributes and affected physiological pathways, demanding comprehensive knowledge of viral structures and host responses which can be provided by virologists, immunologists and geneticists. Consequently, andrologists, reproductive biologists and urologists can make precise assessment of impacts on male fertility. Such collaboration also facilitates the development of customized therapies, involving pharmacologists, clinicians, and scientists in creating tailored antiviral and hormonal treatments specific to the infection and its fertility effects. The collective effort enables a holistic care approach that extends beyond immediate infection treatment, encompassing longer-term recovery support through measures like nutritional counseling and continual reproductive health assessment. Furthermore, the culture of IPE promotes continuous research and learning, vital for enhancing detection and treatment strategies.

Progression of research on male infertility encompasses more than merely interdisciplinary cooperation; it also pertains to societal understanding. The conventional perspective that primarily attributes infertility to females warrants a re-evaluation in the foundational framework. By laying importance on the studies that underscore the frequency and etiology of male infertility, the medical fraternity can significantly influence a change in societal viewpoints, consequently diminishing the associated stigma experienced by numerous men.

### Bridging and compensation of knowledge gaps by interprofessional collaboration: clinical implications

Urologists possess an extensive knowledge of the anatomical and physiological factors related to male infertility. However, they might not be fully versed in the complex genetic components that play a role in this condition (Poongothai et al., 2009[5]). On the other hand, geneticists might benefit from a more expansive grasp of the endocrine dynamics influencing sperm functionality and production. IPE allows these specialists to attain a holistic understanding of the condition of a patient, facilitating more precise diagnoses and tailored therapeutic strategies. Furthermore, incorporating mental health experts and sociologists into the discourse provides an in-depth perspective on the psychosocial ramifications of male infertility (Dooley et al., 2014[3]). This component, while sometimes underestimated, is vital for a comprehensive approach to patient care. By comprehending the psychological repercussions of infertility, healthcare professionals are better equipped to provide support to patients, catering to both their physical and emotional hurdles (Dooley et al., 2014[3]).

Integrating biomedical researchers into this interprofessional paradigm can also streamline the transition of laboratory investigations to clinical applications (Thistlethwaite, 2012[9]). Establishing a seamless dialogue between fundamental researchers and practicing clinicians expedites the genesis of groundbreaking diagnostic and therapeutic methods, ensuring rapid delivery of these advancements to those in dire need. However, the application of an IPE framework in male infertility research is accompanied by hurdles. This method demands substantial modifications to the existing educational and professional paradigms, which are often compartmentalized. Practical issues, like synchronizing timelines and fostering efficient communication among professionals from varying domains, also arise. Yet, the potential gains from this methodology in enhancing our comprehension and treatment of male infertility are too consequential to sideline.

Therefore, the adoption of an IPE strategy offers immense potential in male infertility research for more nuanced diagnoses, improved therapeutic interventions, and holistic patient care. As we navigate this evolving landscape of medical research, harnessing the expertise of all specialties becomes essential to address the multifaceted challenges of male infertility comprehensively.

## Declaration

### Funding

None.

### Conflict of interest

None.
